# Sendai Virus Induces Persistent Olfactory Dysfunction in a Murine Model of PVOD via Effects on Apoptosis, Cell Proliferation, and Response to Odorants

**DOI:** 10.1371/journal.pone.0159033

**Published:** 2016-07-18

**Authors:** Jun Tian, Jayant M. Pinto, Xiaolan Cui, Henghui Zhang, Li Li, Yulong Liu, Chan Wu, Yongxiang Wei

**Affiliations:** 1 Department of Otolaryngology Head & Neck Surgery, The First Hospital of Shanxi Medical University, Shanxi Medical University, Taiyuan, Shanxi Province, China; 2 Section of Otolaryngology-Head and Neck Surgery, Department of Surgery, The University of Chicago, Chicago, Illinois, United States of America; 3 Institute of Chinese Materia Medica, China Academy of Chinese Medical Sciences, Chaoyang District, Beijing, China; 4 Peking University People’s Hospital, Peking University Hepatology Institute, Beijing, China; 5 Department of Otolaryngology Head & Neck Surgery, Beijing Chaoyang Hospital, Capital Medical University, Beijing, China; 6 Department of Otolaryngology Head & Neck Surgery, Beijing Anzhen Hospital, Capital Medical University, Beijing, China; Duke University, UNITED STATES

## Abstract

**Background:**

Viral infection is a common cause of olfactory dysfunction. The complexities of studying post-viral olfactory loss in humans have impaired further progress in understanding the underlying mechanism. Recently, evidence from clinical studies has implicated Parainfluenza virus 3 as a causal agent. An animal model of post viral olfactory disorders (PVOD) would allow better understanding of disease pathogenesis and represent a major advance in the field.

**Objective:**

To develop a mouse model of PVOD by evaluating the effects of Sendai virus (SeV), the murine counterpart of Parainfluenza virus, on olfactory function and regenerative ability of the olfactory epithelium.

**Methods:**

C57BL/6 mice (6–8 months old) were inoculated intranasally with SeV or ultraviolet (UV)-inactivated virus (UV-SeV). On days 3, 10, 15, 30 and 60 post-infection, olfactory epithelium was harvested and analyzed by histopathology and immunohistochemical detection of S-phase nuclei. We also measured apoptosis by TUNEL assay and viral load by real-time PCR. The buried food test (BFT) was used to measure olfactory function of mice at day 60. In parallel, cultured murine olfactory sensory neurons (OSNs) infected with SeV or UV-SeV were tested for odorant-mixture response by measuring changes in intracellular calcium concentrations indicated by fura-4 AM assay.

**Results:**

Mice infected with SeV suffered from olfactory dysfunction, peaking on day 15, with no loss observed with UV-SeV. At 60 days, four out of 12 mice infected with SeV still had not recovered, with continued normal function in controls. Viral copies of SeV persisted in both the olfactory epithelium (OE) and the olfactory bulb (OB) for at least 60 days. At day 10 and after, both unit length labeling index (ULLI) of apoptosis and ULLI of proliferation in the SeV group was markedly less than the UV-SeV group. In primary cultured OSNs infected by SeV, the percentage of cells responding to mixed odors was markedly lower in the SeV group compared to UV-SeV (*P* = 0.007).

**Conclusion:**

We demonstrate that SeV impairs olfaction, persists in OE and OB tissue, reduces their regenerative ability, and impairs the normal physiological function of OSNs without gross cytopathology. This mouse model shares key features of human post-viral olfactory loss, supporting its future use in studies of PVOD. Further testing and development of this model should allow us to clarify the pathophysiology of PVOD.

## Introduction

Post viral olfactory disorders (PVOD) constitute a major category of olfactory impairment [1]. For unknown reasons, in some patients, olfactory loss persists after the upper respiratory tract infection (UTRI) symptoms resolve, despite a lack of residual nasal congestion or obvious sinonasal pathology [2]. Due to the complexity of the underlying physiology, PVOD is difficult to study in humans. Thus, there are no known treatments of this burdensome condition.

Because the onset of olfactory loss presumably occurs secondary to the viral insult, a direct damage of the olfactory sensory neurons (OSNs) is likely. OSNs are unique in their ability to undergo cellular turnover and replacement throughout life [3]. Thus, if we could understand how specific viral infections block this process, we could begin to design therapies to alleviate this condition. The development of a mouse model of PVOD would represent an advance for the field, allowing systematic inquiries into specific viral agents, mechanisms, and potential therapies.

Recently, several studies [4–7] found that there is a higher detection rate of Parainfluenza virus type 3 in patients with PVOD compared with controls. However, subsequent attempts at developing animal models of PVOD using this agent have been challenging, mainly due to the poor ability of this virus to infect murine nasal epithelium. However, in 1984, Kristensson et. al [8] observed that viral antigens appeared in the olfactory nerve axon, neurons and peripheral part of the olfactory bulbs in newborn mice by immunoperoxidase technique following intranasal instillation of Sendai virus (SeV). This virus is a murine counterpart of the human Parainfluenza virus, and has been used extensively in studies that have defined most of the basic biochemical and molecular biologic properties of the paramyxoviruses [9], a class to which they both belong. After 6 days, acute infection appeared to subside, and after 12 days virus antigen was seen only in a few cells in the olfactory epithelium. In 1995, Mori et. al [10] detected the SeV nucleoprotein gene in the olfactory bulbs of intranasally infected mice for at least 168 days post infection (p.i.) by polymerase chain reaction (PCR). Recently, Klemens et. al [11] developed a murine model of viral rhinosinusitis using SeV. After acute infection resolved spontaneously within 10 days, infected mice developed a significant increase in T-suppressor and T-regulatory cells, which persisted for at least 38 days. All of these results demonstrate that paramyxoviruses affect nasal disease, can directly infect olfactory neurons and can establish long-term persistence of viral proteins in the neural tissue. Unfortunately, none of these studies evaluated viral effects on olfactory function itself.

Furthermore, parainfluenza viruses have been found to show anti-apoptotic activity in cultured cells [12], but do not spread throughout the central nervous system (CNS), consistent with the clinical picture of PVOD and, unlike other neurotropic viruses that can infect OSNs without inducing apoptosis, reach the olfactory bulb and spread in the CNS transneuronally. This class is also different from some (but not all) influenza viruses that induce apoptosis in OSNs upon infection and block virus invasion into the olfactory bulb [13]. Such differences might explain why Parainfluenza virus may be a major cause of PVOD.

In this study, we developed a murine model to study the effects of SeV on olfactory function *in vivo* and *in vitro* and tried to illuminate whether the persistence of SeV in OSNs would impair the normal physiological functions of host cell without gross cytopathology. We hypothesized that the anti-apoptotic activity of SeV would keep infected dysfunctional cells alive long and reduce the regeneration ability of the normal olfactory epithelium, blocking recovery of olfactory function. To address this, we detected the status of apoptosis and proliferation in olfactory epithelium *in vivo* in our mouse model of PVOD.

## Materials and Methods

### Study Design

Wild-type, 6- to 8-week-old, male, pathogen-free C57BL/6 mice (n = 120) (17–22g, Vital River Laboratory Animal Technology Co. Ltd., Beijing, China) were handled in accordance with Government of China and US National Institutes of Health guidelines and maintained in the biological safety laboratory of the Institute of Chinese Materia Medica, China Academy of Chinese Medical Sciences, Beijing, in microisolator-top cages. The studies were approved by the Animal Care and Use Committee of the Capital Medical University, Beijing.

Animals were randomly assigned to the experimental and control groups. Each group was then divided into 5 subgroups (n = 12 each) according to time to sacrifice after virus infection (days 3, 10, 15, 30 and 60 after inoculation), which was determined by duration of acute symptoms and the virus replication in nasal cavity [11]. In each subgroup, half the animals were used for coronal section histopathology and the other half for nucleic acid isolation.

To determine whether olfactory dysfunction persisted after resolution of acute symptoms, olfactory function of mice in the subgroup 5 (sacrificed at 60 days) were assessed at each time point prior to sacrifice.

### Viral Inoculation

Sendai virus 52 strain (American type culture collection [ATCC^®^] VR-105^™^) was employed in these experiments. Tissue culture infective dose 50 (TCID[50]) was used to calculate SeV infectious titer,(TCID[50] is defined as the amount of virus required to infect 50% of monolayers of cultured LLC-MK2 cells) [14]. After a transient ether inhalational anesthetic, the experimental group mice were infected with 40μL of cell culture medium contained 10^4^ TCID(50)/mL SeV delivered intranasally on day 0. The control groups were treated by Ultraviolet (UV)-inactivated virus, which was prepared by placement of an aliquot of the inoculant beneath a UV lamp within a class II laminar-flow hood for 1 hour.

Mice infected with SeV were maintained in a negative pressure isolator. Manipulations of the mice were performed within the negative-pressure isolator within class II laminar-flow hoods.

### Olfactory Assessment

The buried food test (BFT), a simple behavioral assessment of mouse olfaction [15], was used to measure olfactory function of mice, the data of subgroup 5 (sacrifice at day 60) was used for analysis for convenience. Twenty-four hours before the test, all chow pellets were removed from the food hopper of the home cage; the water bottle remained. Then, one mouse was placed in a larger cage containing an approximately 4-g pellet of rodent food buried under approximately 3 cm of Sani-Chips bedding. The time (in seconds) that the mouse required to uncover the food and grab it in its forepaws or teeth was recorded. The mouse was allowed to consume a portion of the pellet and then was returned to its home cage. Each animal was tasked in the same manner and underwent the same test 3 times, with the food hidden in a new position chosen for each round of trials. The food-restricted mice which failed to use odor cues to locate the food within a 6 minute period were defined to have deficits in olfactory abilities.

### Histopathology and immunohistochemical detection of S-phase Nuclei and Apoptosis

Mice were sacrificed by CO_2_ asphyxiation, and after decapitation, the skin and soft tissue were removed from the skull, lower jaws were removed, and the remaining part of the heads were fixed with 4% paraformaldehyde in 0.1 M phosphate buffer (pH 7.4) for 48 hours. They were then decalcified with10% EDTA in TRIS buffer (pH 7.6) for 14 days, following rinsed overnight in running tap water and further fixation for 24 hours in 4% paraformaldehyde. Level III sections were trimmed, dehydrated, embedded in paraffin and sectioned coronally at a thickness of 7μm. Sections were collected and placed on silane-coated slides. [11, 16] Additionally, the structure and Integrity of the olfactory epithelium was demonstrated using hematoxylin and eosin staining (H&E staining) and immunohistochemistry by olfactory marker protein (OMP) (1:200) (Abcam Hong Kong Ltd, Hong Kong). Polyclonal antibody to Sendai virus was used to identify the cells infected (1:200) (Abcam Hong Kong Ltd, Hong Kong). The isotype controls came from homologous serum. An Aperio Scanscope AT Turbo (Aperio Technologies, USA) was used to capture the images. Then, these sections were used for histopathology and immunohisochemical detection of S-phase nuclei and apoptosis according the protocols below.

### OSN Proliferation

Click-iT^®^ Plus EdU Imaging Kits (Invitrogen, Carlsbad, CA) was used to directly measure OSN DNA synthesis. In contrast to BrdU assays, detection of the incorporated EdU (5-ethynyl-2′-deoxyuridine, a nucleoside analog of thymidine) was more efficient, using standard aldehyde-based fixation and detergent permeabilization. The manufacturer’s protocol was adapted for histological staining of nervous system [17].

**Fig 1:**
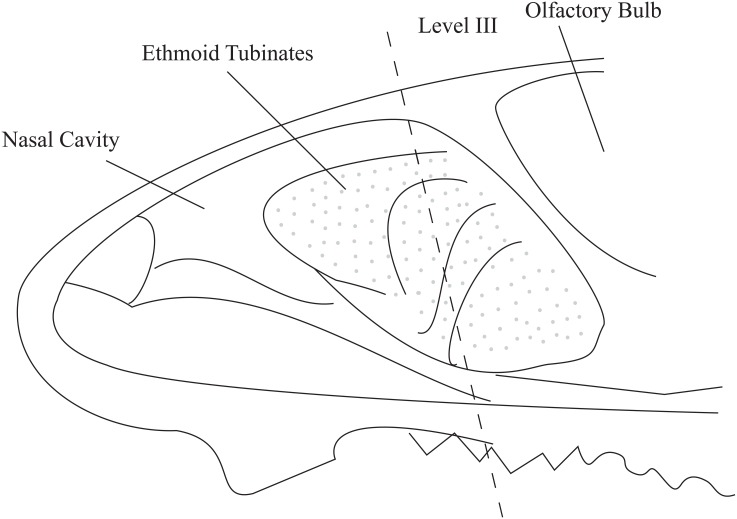
Location of olfactory epithelium in murine nasal cavity and section for histology. The dotted line indicated the location of coronal section at Level III. Shaded areas are the ethmoid turbinates, which are covered by olfactory epithelium.

Four hours prior to sacrifice, mice were administered Edu (200ul of 100mg/kg dissolved in warm physiological saline) in order to label S-phase nuclei. Proliferation of the olfactory epithelial cells was assessed by counting S-phase (i.e., Edu positive) basal cell nuclei (both so-called horizontal and globose basal cells) along the basement membrane. Basement membrane lengths were determined by manual measurements using Image-Pro^®^6.0 (Media Cybernetics, Inc.USA). Unit length labeling index (ULLI) was expressed as the number of S-phase cells per mm of basement membrane [18]. ULLIs were calculated on both sides of the nasal septum as well as lateral ethmoid turbinates of the entire Level III nasal cavity section [16] (Fig 1).

### OSN Apoptosis

TUNEL staining was performed according to manufacturer’s protocol (DeadEnd^™^ Fluorometric TUNEL System, Promega Corporation, USA) for the specific detection and quantitation of apoptotic cells within the olfactory epithelium in the paraformaldehyde-fixed histological section. The system measures the fragmented DNA of apoptotic cells by catalytically incorporating fluorescein-12-dUTP(a) at 3′-OH DNA ends using the Terminal Deoxynucleotidyl Transferase, Recombinant,enzyme (rTdT). OSN apoptosis was assessed by counting apoptosis positive cells along the basement membrane of the olfactory epithelium, as described above for proliferation.

### Viral load by real time PCR

To collect olfactory epithelium for RNA extraction, an incision was made on the skull along the midline and the septal epithelium, olfactory turbinates and bilateral olfactory bulb were dissected free under stereomicroscopy using published procedures [19]. The tissues were immediately put in 1 mL RNA later (RNAlater RNA Stabilization Reagent, QIAGEN, German) and stored at -80°C. Once all samples were collected, total RNA was extracted for each mouse using the TriReagent procedure provided by the manufacturer. The yield, purity, and quality of the total RNA for each sample were determined with a Nanodrop 2000 (Thermo Scientific) and RNA electrophoresis.

Quantification of SeV RNA was determined by Real-Time Reverse Transcriptase–Polymerase Chain Reaction with standard curve (ABI7500 Applied Biosystems, SYBR Green I). Primers for the SeV Haemagglutinin/Neuraminidase (HN) gene had the following sequences: 5' AAAATTACATGGCTAGGAGGGAAAC3/ (sense) and 5' GTGATTGGAATGGTTGTGACTCTTA 3' (antisense). The final product was 104 bp in length. The thermal cycling program was: 95°C for 10 minutes, then 95°C for 15 seconds and 60°C for 60 seconds, for 45 circles total.

### Culture of Primary Mouse Olfactory Sensory Neurons

Primary OSN cultures were prepared from the C57BL/6 embryos between E15 to E20, as described previously [20–21]. Briefly, olfactory mucosa was dissected free from the nasal cavity under stereomicroscopy. After 40 minutes of incubation in 2 mg/ml dispase at 37°C, olfactory epithelial cells were separated from the underlining stroma using fine tungsten needles and the remainder were incubated in Waymouth's MB 752/1 medium with N2 supplement (Invitrogen, USA) at 37°C for 3 hours to allow OSNs to migrate towards the surface of the pseudo-stratified olfactory epithelium. To avoid the contamination of other type of cells in the neuroepithelium, OSNs were dissociated by triturating the tissue for 10–15 times in a 15mL Falcon tube without the intermediate treatment with 0.05% trypsin. For the same reason, dissociated cells were directly plated at a density of 5 × 10^5^ cells/ml on a 35mm glass bottom dish without a confluent astrocyte feeder layer.

OSNs were identified on day 5 by immunofluorescence [22]. The sources and the dilutions of the primary antibodies were: chicken polyclonal antibody to mouse β-tubulin III (1:200) (Abcam Hong Kong Ltd, Hong Kong) and rabbit polyclonal antibody to mouse olfactory marker protein (1:200) (Abcam Hong Kong Ltd, Hong Kong). Standard flow cytometry was used to test the percentage of OSNs. Briefly, primary cultured cells were collected in to three tubes and each tube was then labeled with phosphate-buffered saline only, second antibody-FITC only and β-tubulin III antibody (1:200) (Abcam Hong Kong Ltd, Hong Kong) with second antibody-FITC respectively. Finally, the stained cells were analyzed in a flow cytometer (BD LSRFortessa^™^, San Jose, CA).

### Infection of Cultured OSN with Sendai virus

We observed that olfactory sensory neuron cultures can be maintained for as long as 10 days under these conditions, even without a feeder layer of cortical astrocytes. However, establishing successful cultures was difficult because OSNs are sensitive to external environment and more likely to die *in vitro*. In order to improve OSNs survival rate, the existing culture media was not removed completely; this change in protocol allowed the SeV infection to proceed for 2–3 hrs at 37°C, which was different from usual infection time in the *in vivo* model. Briefly, 6 hours after plating, 50% of the OSN medium was changed with fresh warm medium, and 24 hours later, half of the medium of infection group was replaced with SeV diluted in culture medium at a 200 TCID(50), whereas the control group was changed with fresh warm medium lacking SeV (vehicle). The virus solution was not removed immediately after inoculation. From then on, half of the medium was replaced every other day. At day 1, 3, 5, 7 post-infection, cell appearance was measured under inverted microscopy (LEICA 4000B, Germany). The viability of primary cultured cells was determined by trypan blue staining method using an automated cell counter (Shanghai Ruiyu Biotech Co. Ltd, Shanghai, China).

### Fura-4 Ca2+ Imaging of Odorant Responses

Intracellular calcium imaging induced by odorants is often used to evaluate physiological function of OSNs [23–25]. In this study, primary cultured OSNs (infected with SeV or UV-inactivated virus) were tested two times independently for responses to a odorant mixtures (phenethyl alcohol, isovaleric acid, methyl cyclopentene ketone, and undecalactone,3-methylindole, dissolved in Ringer’s solution at 100 μM each) by measuring changes of intracellular calcium concentrations indicated by fura-4 AM (Molecular Probes R, Life technologies, USA; working concentration: 4μM/L).

At day 4 after primary plating (i.e., on day 3 post infection), loading of cultured OSNs with fura-4 AM of two groups was done at 37°C for 30 minutes The low-toxicity dispersing agent Pluronic^®^ F-127 (Life technologies, working concentration: 0.05%) was used to facilitate cell loading. Fluorescent signal was detected by laser scanning confocal microscope (Leica Microsystems TCS SP5, USA). Images were acquired every 1.3 seconds and analyzed with Leica Advanced Fluorescence Application Suite v. 2.4.1 software, which computes values for the intensities within manually-identified whole OSN regions and generates a graphical output. Exposure times were 50 ms and the light was shuttered between exposures to minimize photobleaching, as described in Rawson et. al [26].

The fluorescence intensity of OSNs in Ringer’s solution was recorded for 1 minute as a baseline. Then, odorant mixtures were added by a computer-controlled solution changer (RSC- 100; Bio-Logic, Pullman, WA, USA) at 300–500 μL/minute. In each group, 2~3 fields were selected and 10 isolated OSNs were measured. Finally, an odor was considered to elicit a response if the fluorescence ratio increased or decreased after odor exposure by more than 2 standard deviations [27].

### Statistical Analysis

Independent-samples t-test or ANOVA, as appropriate, were carried out for comparison of weight, time of Buried food test, viral load and ULLI for each group. Function test data in vitro were analyzed with the Chi-square test. *P* values<0.05 were considered significant. Statistical analyses were performed with SPSS (version 17.0, Chicago, USA).

## Results

### In vivo Studies

#### SeV causes decreased olfactory function

**Fig 2:**
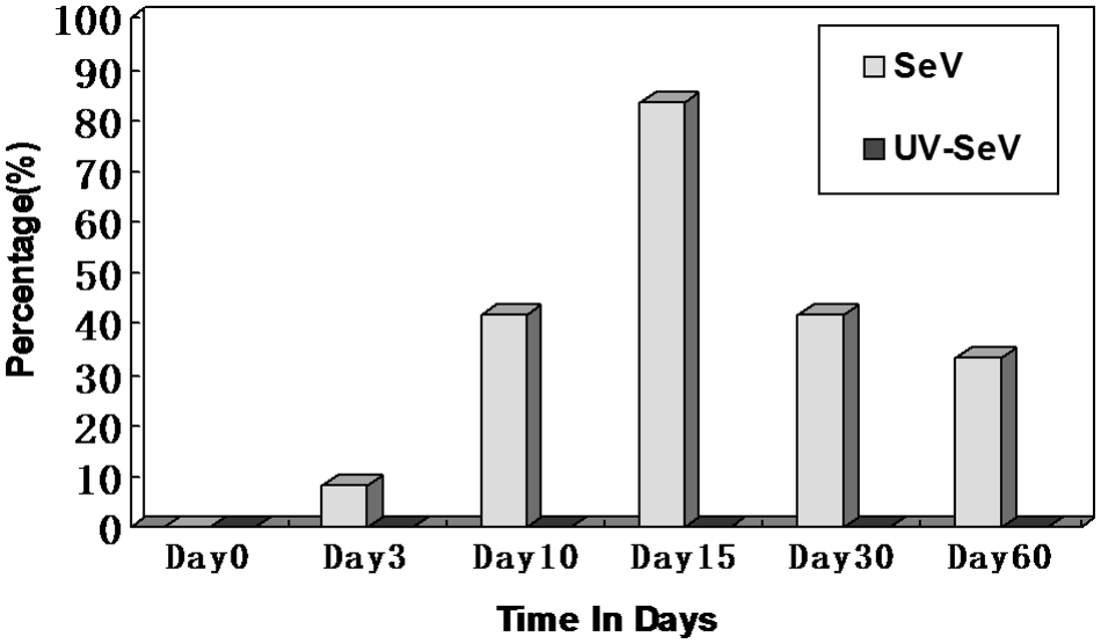
Time course of the incidence of olfactory dysfunction in mice infected with SeV and UV-Inactivated SeV.

Baseline characteristics of the two groups of mice were similar (e.g., weights were 16.8 ± 1.3g vs.16.7 ± 1.5g, SeV vs. UV-SeV, *P* = 0.181). Before incubation, every mouse in group 5 (sacrifice at day 60) was able to find the buried pellet within two minutes, and there was no significant difference between the mean time to find the pellet between the experimental and control groups (SeV 71.3 ± 31.2s vs. 54.3 ± 18.4s vs. UV-SeV, *P* = 0.118). In the SeV group, some of the mice suffered from olfactory dysfunction, peaking on day 15. Two months later, four out of 12 still had not recovered, while in the UV-SeV mice, none lost their olfactory function (Fig 2). On day 30 and 60, the time of the animals to locate the food in the SeV group which did not meet the definition of olfactory dysfunction were longer compared with control group (207.3±46.9 vs. 80.5±23.8, 191.2±52.9 vs. 78.9±24.2, P = 0.001,P = 0.001, respectively). This suggested they suffered at least a partial injury of the olfactory system.

#### Viral load increased after SeV infection

**Fig 3:**
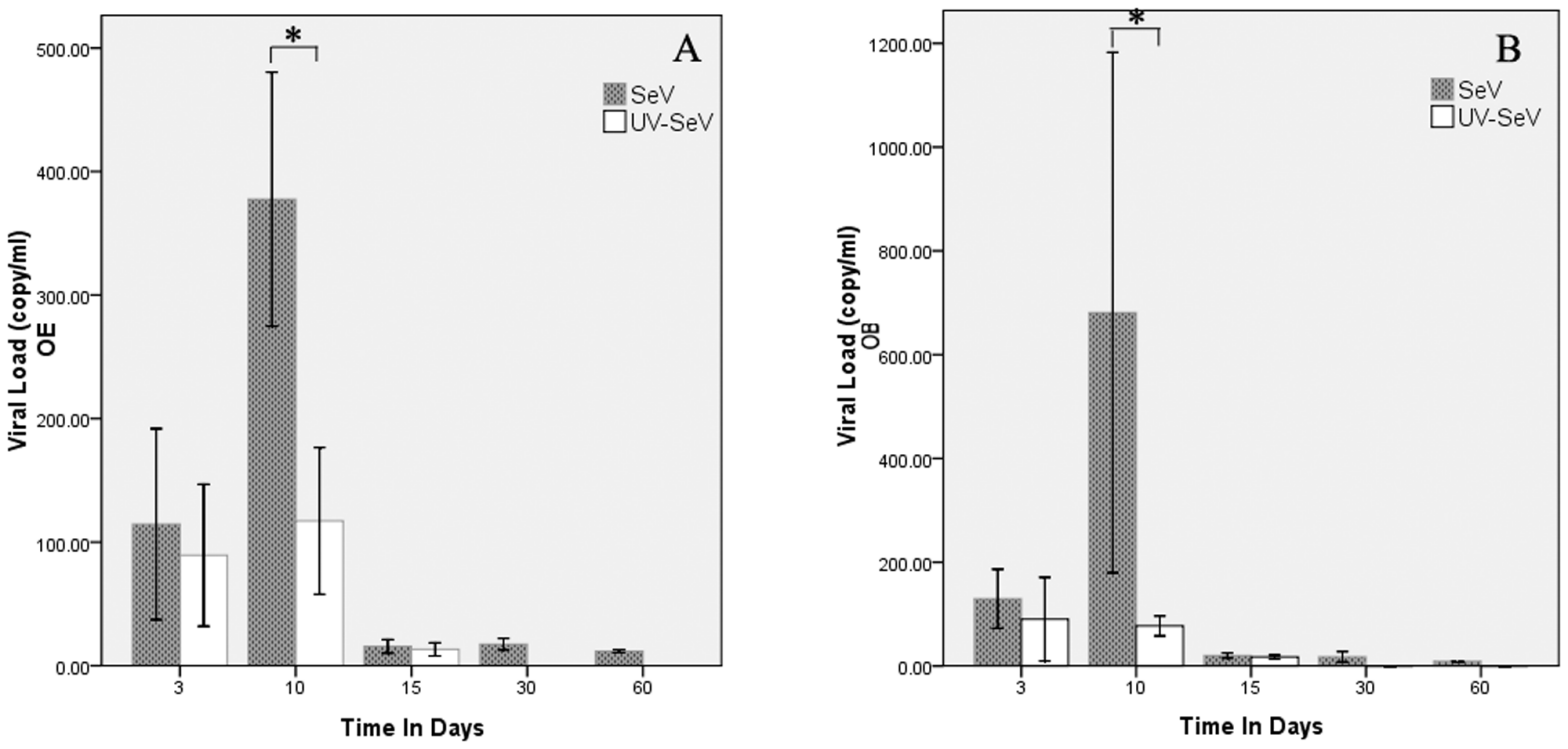
Viral load of SeV by realtime RT-PCR over time. (A) Olfactory Bulb. (B) Olfactory Epithelium.

During observation, virus growth peaked in olfactory bulb and olfactory epithelium at 10 days post-infection (p.i.), and was significantly higher than in the UV-SeV group (*p* = 0.027, *p* = 0.003, respectively). Thereafter, viral load was low, at least by 60 days (Fig 3). In the UV-SeV group, the quantity of viral RNA continued to decline after inoculation, dropping to undetectable levels in both olfactory bulbs and epithelia at 30 days and after.

#### Structural changes in the olfactory epithelium after SeV infection

**Fig 4:**
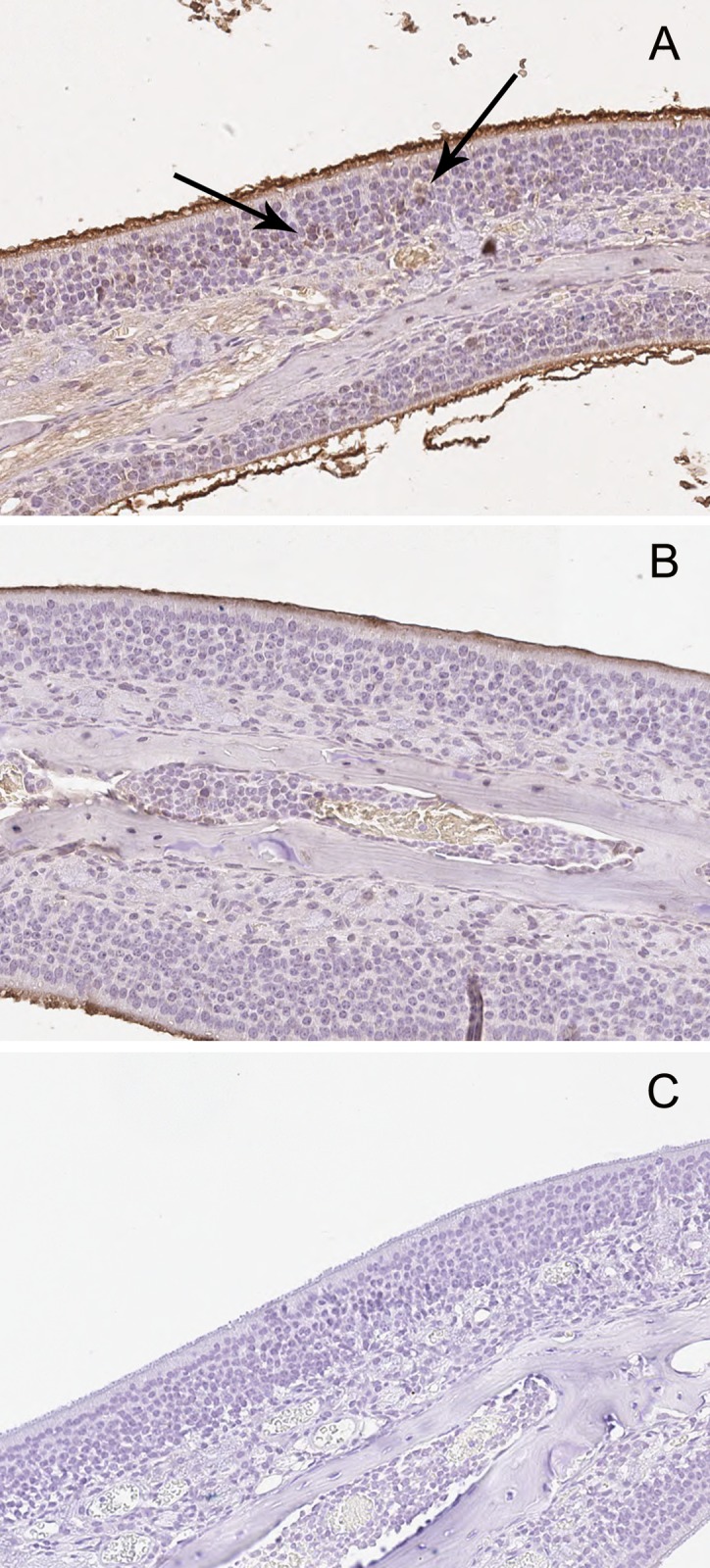
Immunohistochemical detection of Sendai virus proteins in the olfactory epithelium 3 days post-infection. The virus-specific staining was observed in the cytoplasm of some olfactory neurons (arrows) in SeV group (A) but none in UV-SeV group (B). (C) Isotype control (200X).

**Fig 5:**
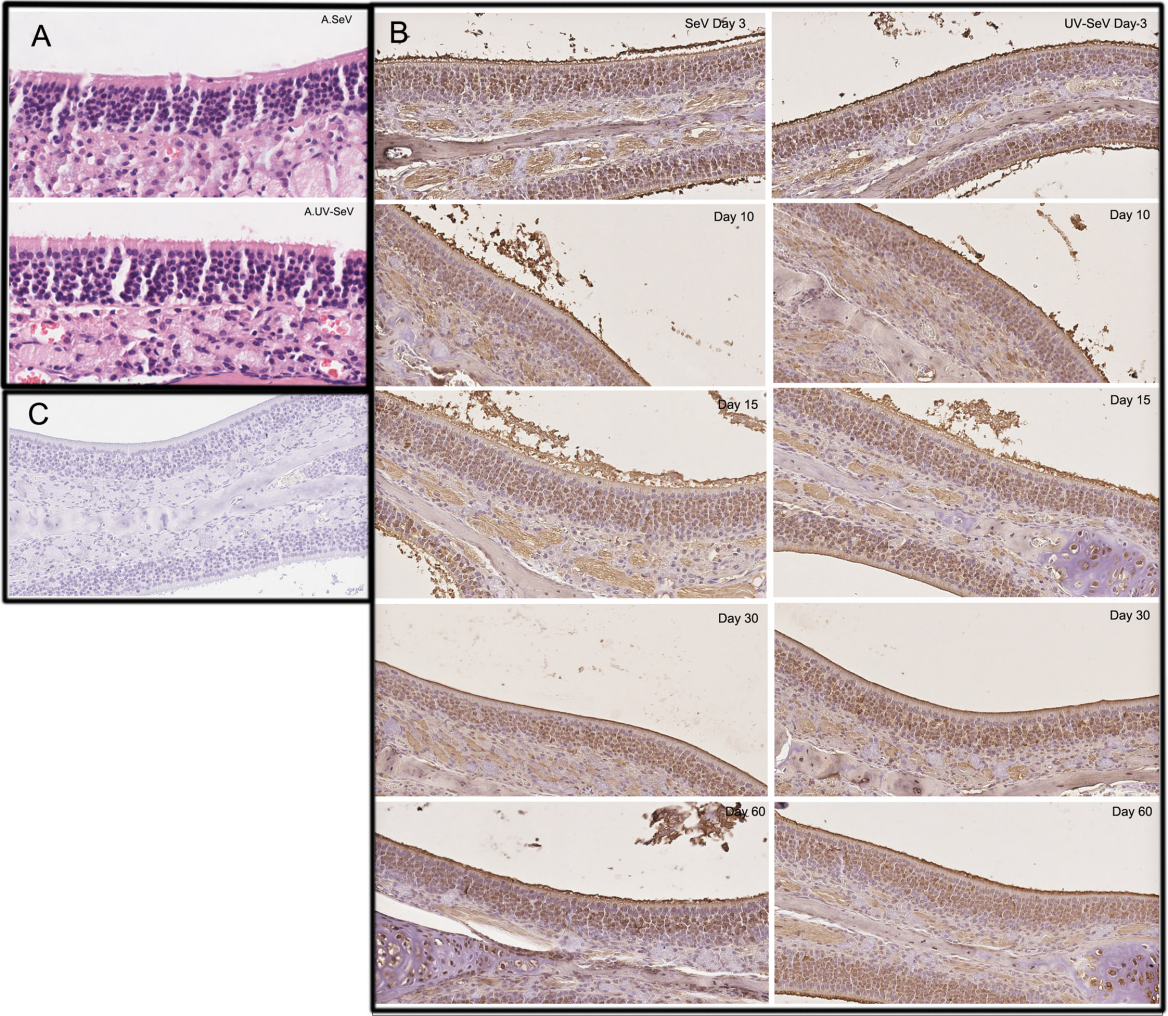
Histological changes by hematoxylin-eosin staining (HE) and immunohistochemical detection of olfactory marker protein (OMP) in the olfactory epithelium post Sendai virus infection. (A) HE staining showed that there is no obvious loss of integrity of the olfactory epithelium in SeV group compared with UV-SeV group 10 days after infection. Macrophages, neutrophils and lymphocytic infiltration in the olfactory mucosa stained with hematoxylin-eosin is not evident. (400X) (B)Immunohistochemical detection of OMP demonstrated that there is no obvious loss of integrity of the olfactory epithelium in both two groups at each time point. (200X) (C)Isotype control (200X).

At 3 and 10 days p.i., Sendai virus F proteins were detected in the cytoplasm of some olfactory neurons of all samples in SeV group (Fig 4A) but in none of the mice in the UV-SeV group (Fig 4B). At 15 days p.i. and subsequent time points, they were not found in any of the samples. By both histology and immunohistochemistry, there was no obvious loss of integrity of the olfactory epithelium in both groups at any time point (Fig 5A, 5B and 5C). Macrophages, neutrophils and lymphocytic infiltration in the olfactory mucosa were not prominent through the course of infection.

#### Decreased apoptosis and cell proliferation after SeV

**Fig 6:**
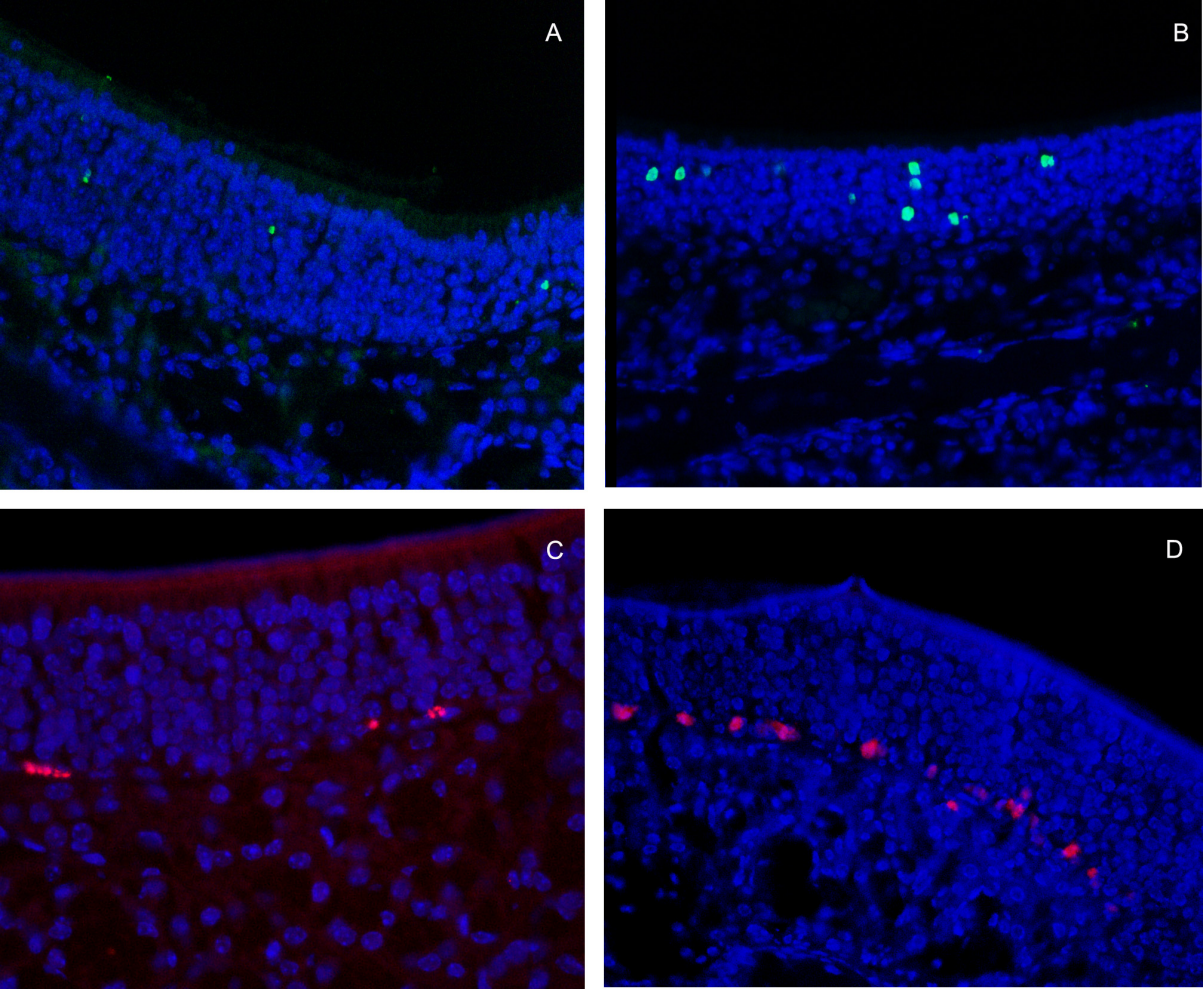
Apoptosis and proliferation of cells in the olfactory epithelium at Day 15 post-infection (200X). Apoptotic cells are marked by green fluorescence mainly located in the middle layer of olfactory epithelium. (A) SeV group. (B) UV-SeV group. Cell proliferation (DNA synthesis marked by EdU) in the olfactory epithelium was located in the basal layer, labeled by red fluorescence. (C) SeV group (D) UV-SeV group.

**Fig 7:**
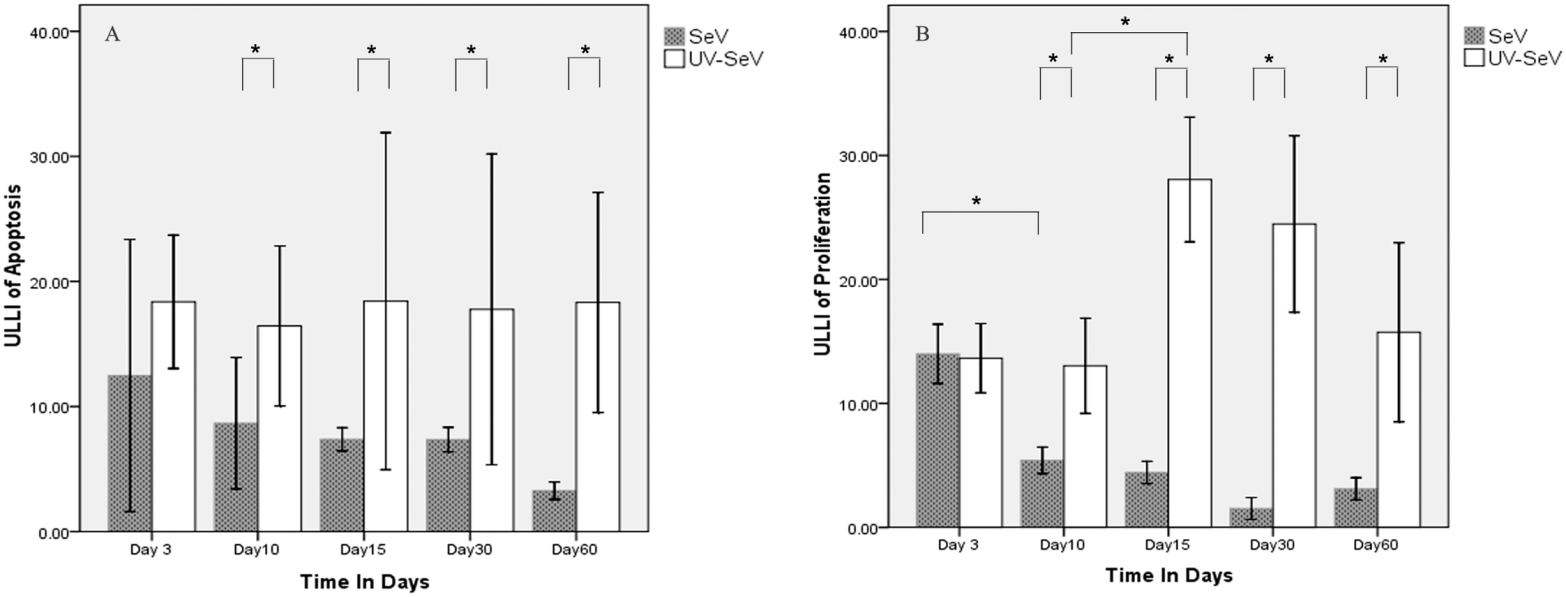
Unit length labeling index (ULLI) of apoptosis and proliferation in olfactory epithelium in mice infected with SeV or UV- SeV. (A) Apoptosis(B)Proliferation (Unit/mm).

Apoptotic cells were found in both groups, mainly located in the middle layer of olfactory epithelium (Fig 6A and 6B). With SeV, ULLI tended to decrease with time, while ULLI in the UV-SeV group remaining unchanged (Fig 7A, *P* = 0.994,S1 File). At day 10 and after, the ULLI in the SeV group was markedly less than the UV-SeV group (*P* = 0.031, *P* = 0.114, *P* = 0.049, *P* = 0.001 respectively). In preliminary experiments, the buried food test and **immunohistochemistry** were performed in six 6 to 8 week old C57BL/6 male mice as a normal control. The olfactory function was normal and the ULLI of apoptosis and proliferation of this group were 17.6±3.0 and 16.7±4.1 respectively.

All the proliferating cells in the olfactory epithelium were located in the basal layer, labeled by red fluorescence (Fig 6C and 6D). Additionally, certain Bowman gland cells in the stroma, and some bone marrow cells under the mucosa were in the proliferative state. A significant reduction of ULLI with time was observed from day 3 to 30 after SeV treatment (Fig 7B, S1 File). At day 10 and after, the ULLI of the SeV group was significantly less than that of the UV-SeV group (*P* = 0.001, *P* = 0.001, *P* = 0.001, *P* = 0.008 respectively). At 60 days p.i., the proliferation was restored partially (*P* = 0.008). In the UV- SeV group, the quantity of proliferating cells increased obviously at 15 days p.i. (*P* = 0.001).

### *In vitro* Studies

#### SeV causes minimal cytopathic effects on OSNs

**Fig 8:**
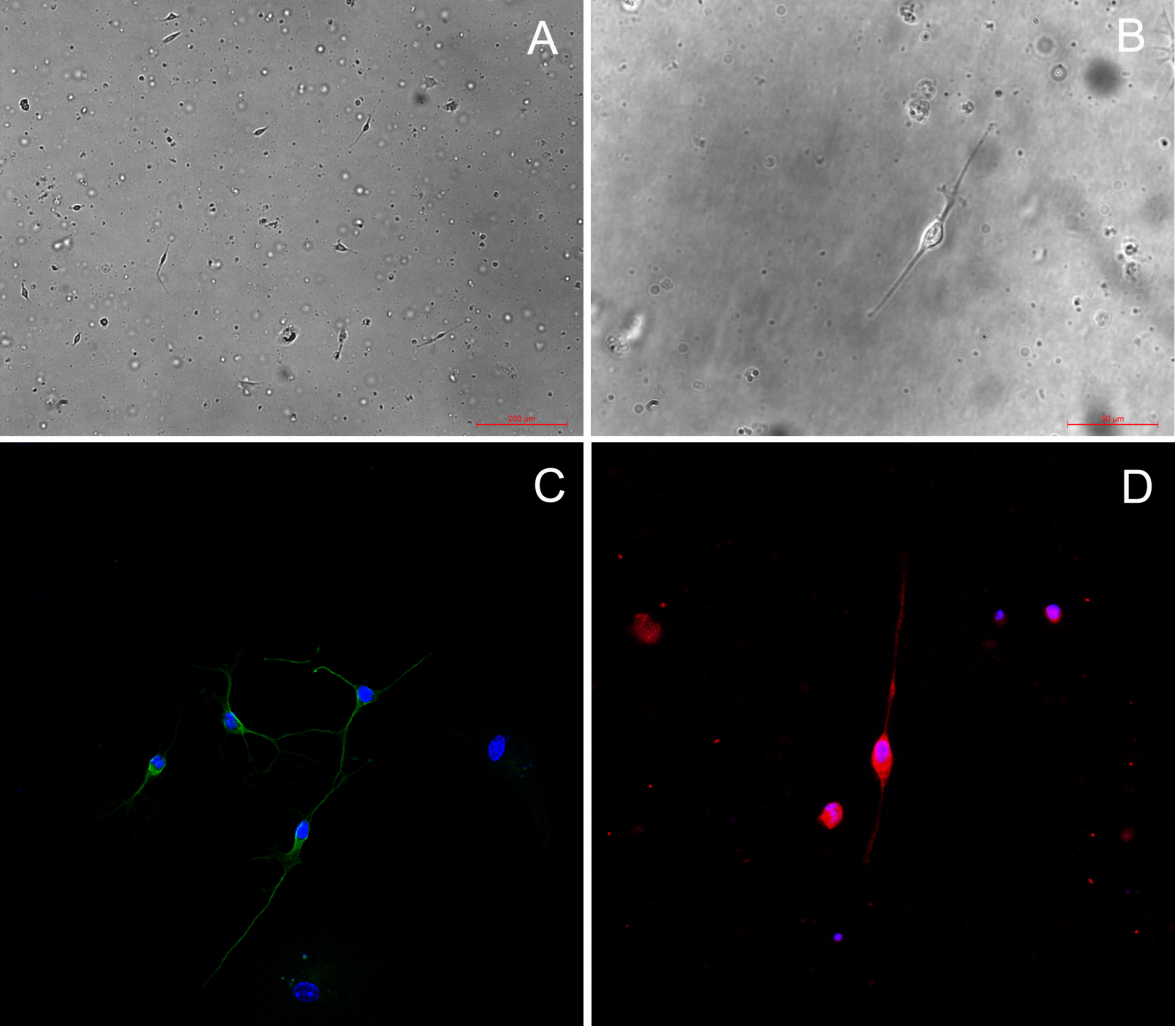
Morphology and identification of primary cultured olfactory neurons (OSNs). (A) At 24 hour after plating, some cultured cells had abnormal morphology, whereas others resembled classical OSNs. (40X). (B) Classical OSNs under high-powered magnification exhibit bipoloar morphology with a round cell body, a small thin process suggestive of an axon, and a short, thick process, suggestive of a dendrite (400X). (C) At 3 days, OSNs are identified by expression of type III β-tubulin under confocal laser microscopy, Synaptic connections between neurons were demonstrated. (800X). (D) At 7 days, some cultured neurons expressed neuronal markers (OMP). (800X).

**Fig 9:**
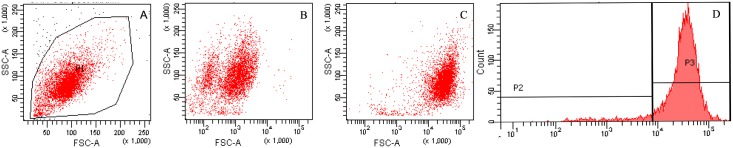
Counts of β-tubulin III positive cells in primary cultured olfactory neurons by flow cytometry. (A) Counts of β-tubulin III positive cells treated with phosphate-buffered saline only. (B) Counts of β-tubulin III positive cells treated with second antibody-FITC. (C)Counts of β-tubulin III positive cells labeled with β-tubulin III antibody (1:200) and second antibody-FITC. (D)Histogram demonstrate the β-tubulin III positive cells (P3), which represents the olfactory neurons.

During the first 24 hours, there was a large amount of cell death. At 24 hour after plating, some cells had abnormal morphology (Fig 8A), whereas others resembled classical OSNs with a round cell body, a small thin process suggestive of an axon, and a short, thick process, suggestive of a dendrite (Fig 8B). OSN were identified by expression of type III β-tubulin and OMP using immunocytochemistry. At 3 days in vitro (DIV), β-tubulin III expression was detected (Fig 8C) and the percentage of positive cells was 90.7% measured by flow cytometry (Fig 9). The percentage of OSNs based on morphology was 32.5%±4.4%. At 7 DIV, OMP positive cells (46.8%±9.2%) appeared in the culture while OMP expression was not detected before this time (Fig 8D). Under confocal laser scanning microscopy, synaptic connections between neurons were demonstrated, olfactory cilia could be distinguished in some OSNs, and bipolar with longer processes could be seen prominently. Although the survival rate was approximately 20–30% of the plated cells between 3 to 5 DIV and plating on non-cellular substrates supported only limited neuronal survival, the surviving OSNs retained their bipolar morphology and were stable at least for 10 days using this protocol.

**Fig 10:**
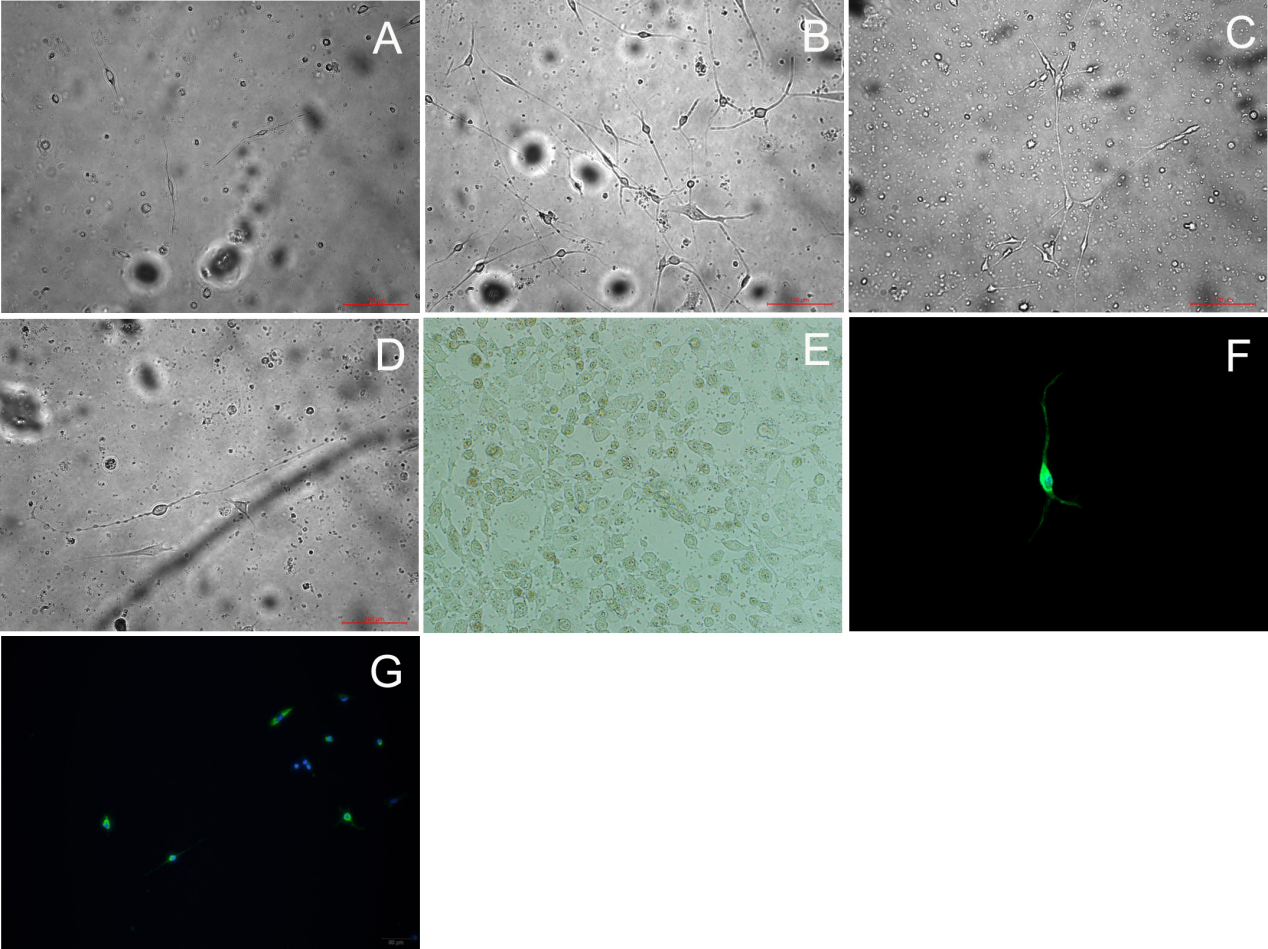
Morphology changes of olfactory neurons in vitro after SeV infection. (A) OSNs treated with UV-SeV at day 3 p.i. (200X). (B) OSNs treated with SeV at day 3 p.i. (200X). (C) OSNs at day 5 p.i. (200X). (D) Single OSN treated with SeV at day 5 p.i. demonstrates a normal bipolar morphology with a long axon under high-power. (400X). (E) Cell-to-cell fusion was observed in the LLC-MK2 cell lines treated with SeV at day 5 p.i. (400X). (F) and (G) SeV F protein was localized mainly in the OSN cell body at day 5 p.i. under confocal laser microscopy. (1:100 MAb dilution, 800X, 200X respectively).

Through continuous observation under inverted microscope, SeV infection of primary cultured OSNs caused minimal gross cytopathology at days 3 and 5 p.i. (Fig 10B, 10C and 10D), with no evidence of cell-to-cell fusion, as seen with infection of non-polarized cell lines like LLC-MK2 (Fig 10E). Compared with the control group (Fig 10A), inoculation with SeV did not increase cell death and the viability of primary cultured cells was 24.1±2.9% and 25.8±2.6% respectively at day 3 p.i. (*P* = 0.348). The typical neuron morphology (round cell body, dendrite and long axon) remained. The SeV F protein was found to be localized mainly in the cell body, as shown by immunofluorescence (Fig 10F and 10G). The average fraction of OSNs infected with SeV from ten isolated fields was 86.8±7.8%, and these cells were neurons based on morphology.

#### Functional responses of OSNs to SeV

**Fig 11:**
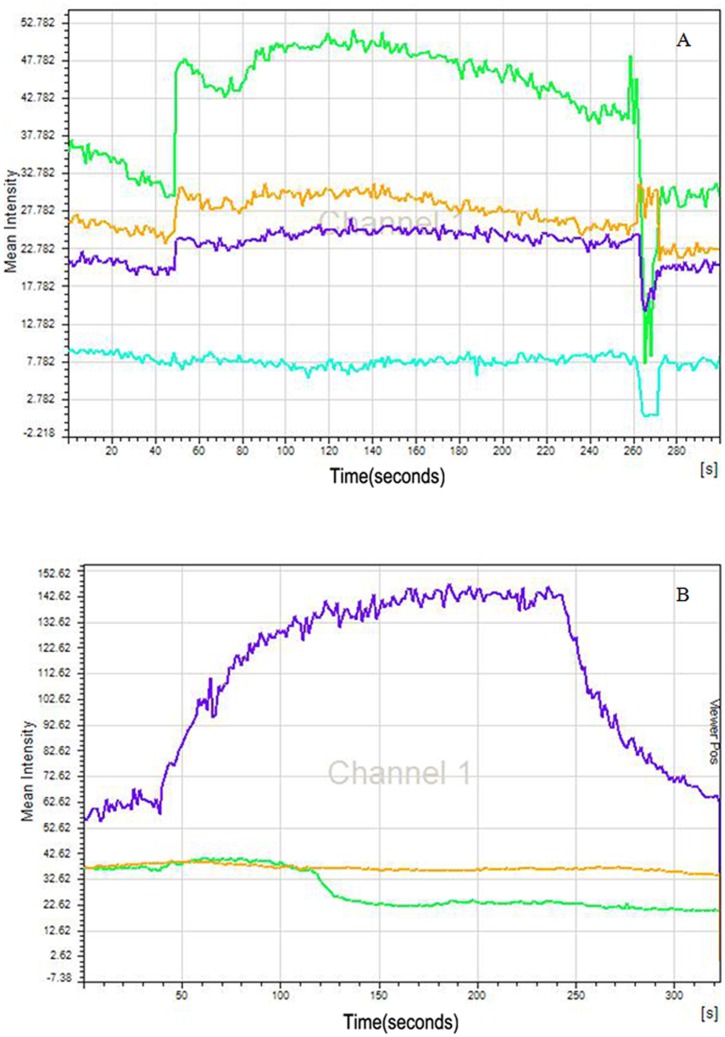
Ca^2+^ responses of primary cultured OSNs after Sendai virus infection induced by olfactory stimulation. (A) The OSNs in the control group whose fura-4 intensity is displayed in green and purple showed increases in intracellular calcium when stimulated with odor mixture that returned to basal levels after removal of the odors. The brown curve represented a possible untypical olfactory neuron without the bipolar morphology but having the similar cell body with OSNs, which nevertheless had the same response. The bottom curve was recorded from another type of cell with a large nucleus meaning fibroblast and shows no responses. (B) SeV infected OSN did not show calcium Ca^2+^ influx upon stimulation with odor, displayed in green and brown curve. The purple curve represented a cell without typical OSN morphology in the SeV group but having the similar cell body with OSNs, and shows a slow but continuous influx of Ca^2+^ induced by mixed odors and demonstrate a slow decreasing after withdraw of stimulation, which suggests a bit dysfunction in the regulation of cytosolic Ca^2+^.

To determine whether OSN infected with SeV retain the ability to function, an odorant response assay was performed by fluorescence intensity using fura-4 imaging two times independently. For the first time, in UV-SeV treated cells, 80% of OSNs identified by typical bipolar morphology showed immediately increases in intracellular Ca^2+^ levels in response to mixed odorant stimulation, and returned to baseline after removal of the odor (addition of Ringer’s) (Fig 11A). In contrast, stimulation with same odorants resulted in no change in fluorescence intensity in 90 percent of OSNs treated with SeV (Fig 11B). The percentage of cells responding to mixed odors was markedly lower in the SeV group than in the control group (10% vs. 90%, *P* = 0.007). In another separate repeated experiment, the percentage was 20% vs. 90% (*P* = 0.005). Additionally, in the SeV group, some of cells that have responses showed a slow but continuous influx of Ca+ induced by mixed odors and demonstrated a slow decreasing after withdraw of stimulation, which suggests some dysfunction in the regulation of cytosolic Ca^2+^. However, the fluorescence intensity in responding cells of control group that had been exposed to olfactory stimuli rapidly increased once odors added and remained high until the odors were removed.

## Discussion

In this study, we have shown that 6–8 week old C57BL/6 mice suffered from impaired olfaction following intranasal infection with SeV, a deficit that persisted at least for 2 months in approximately 33% of animals. These effects were mirrored by increases in viral load, decreased apoptosis and decreased cell proliferation. Thus, this model recapitulates the key clinical features of human PVOD to a reasonable degree. These data suggest that SeV infection suppresses the apoptosis of OSNs, impairing the normal regenerative ability of the olfactory epithelium. This may be one explanation, among others, of why recovery of olfactory function after viral infection of OSNs occurs.

To determine a mechanism of these effects, we utilized an *in vitro* system to study the functional effects of SeV infection in OSNs. In SeV infected primary OSNs cultures, we found that the ability of the olfactory cells to take up Ca^2+^ after stimulation by mixed odorants was significantly reduced compared with control. Interestingly, we found no obvious cytopathic effect of SeV infection. These results suggest that SeV itself might alter OSN function directly, in addition to the effects on cell turnover via indirect effects.

It is known that the viral infection is a common cause of clinical olfactory disorders [28]. In the early phase of URTI, a conductive or obstructive loss secondary to mucosal edema is a major mechanism of olfactory loss. In some patients, this loss will persist after the cold symptoms resolved. At this point, it is possible that the loss becomes sensorineural secondary to the viral insult. Although research is progressing rapidly in the basic science of human olfactory physiology, our understanding of clinical disease is minimal, especially for PVOD. Besides the challenges of studying olfactory disorders in humans, one of the most important reasons for this situation is that the causative viruses have not been easy to identify, nor do we understand the mechanism of the insult.

Parainfluenza virus 3 has been implicated in PVOD. SeV is a counterpart of human Parainfluenza virus. Animal models of SeV-related respiratory diseases have been established many decade years ago for other disorders. In 1984, studies of Kristensson et. al [8] demonstrated that SeV can infect respiratory epithelium and olfactory mucosa in newborn mice following intranasal instillation. In 1995, another study by Mori et. al [10] found that the SeV genome persisted in the olfactory neurons but not lungs in the adult mice for a long period, and that infected mice showed mild respiratory symptoms but displayed no appreciable neurological symptoms. Unfortunately, the authors did not disclose which kind of neurological symptoms they assessed.

We confirmed this persistence, but also demonstrated olfactory problems *in vivo* and *in vitro*, which may aid further studies about the relations between SeV and OSNs. In these experiments, the viral load of SeV peaked at day 10 after infection. These results differ from the work of others [10, 29–30], which showed that virus growth peaked in nasal turbinates, tracheas, and lungs at 5 days p.i., but may be related to difference in protocol, assessment time, and location (we did not examine turbinates or the lower airway). We found that the peak of olfactory dysfunction in the mice appeared at day 15 p.i., a time point at which the immune responses against SeV had geared up for nearly 10 days. This time delay demonstrates a process of impaired olfactory induced by SeV and the host immune response to it, in addition to direct damage to the OSNs themselves.

Generally, Sendai virus infection and replication lead to a strong cytopathic effect on respiratory epithelium with subsequent death of host cells and apoptotic cell death in other airway disease models [9, 31–32]. However, consistent with other *in vivo* studies [8, 10], we found that SeV infection of OSNs causes minimal gross cytopathology both *in vitro* and *in vivo*, with no evidence of cell-to-cell fusion. By contrast, SeV infection of non-polarized cell lines, such as LLC/MK2 cell lines, is characterized by syncytium formation leading to cytopathology. It is reasonable to speculate that SeV maybe have a different effect on the different type of host cells. Just like other members belonging to the genus Respirovirus within the family Paramyxoviridae, infection of non-polarized cell lines in vitro is characterized by syncytium formation leading to cytopathology, while infection of polarized cells causes minimal gross cytopathology [33]. Recently, several studies support this concept. Chambers [34] reported that rSeVhP, in which the SeV P/C gene was replaced with that of HPIV-1, induced apoptotic cell death in murine but not in A549 cells and suggested SeV V protein, possibly in conjunction with the C protein, functions to suppress apoptosis in infected murine cells. Another study [35] showed IRF-3 controlled the fate of the SeV-infected cells by promoting apoptosis and preventing persistence and the levels of expression are highly different in different type of cells. Sendai virus trailer RNA simultaneously blocks two apoptosis-inducing mechanisms in a cell type-dependent manner [36]. What connects blockage of apoptosis and establishment of persistent infection in OSNs remains to be explored.

Some studies [37–38] have found that UV-inactivated Sendai virus is different from a saline control, which is unable to replicate in cells because the genome is destroyed by UV irradiation, but still contains the complete structure of the live HVJ envelope containing F, HN, and M proteins. These proteins have displayed antitumor effect through enhancing the immune responses or inducing apoptosis in a variety of carcinomas. The biological effects of these proteins may be one of the reasons why we observed a relatively high degree of apoptosis even in our control group after UV-SeV treatment. So, we speculate that UV-inactivated Sendai virus has similar effect on the olfactory epithelium in vivo and it might highlight the anti-apoptosis effect of active SeV using an UV-SeV as control. We performed additional experiments with normal saline as a control to determine baseline rates of apoptosis in our experimental setup. These data demonstrated a higher rate of apoptosis in normal control tissue than found in some studies [39–40] (S1 File). There are several potential explanations for this. For example, some studies show that olfactory neurons are vulnerable to toxicity (such from as serine protease inhibitor Spi2, Staphylococcus infection, brief exposure to copper, etc.), and apoptosis of OSNs may be elevated after exposure to these molecules [41–43]. Additionally, in this set of experiments, pellets were removed for 24 hours to test their olfactory function and inhalation anesthesia by 1.5% isoflurane was employed. Several studies [44–45] demonstrated that neurons of mice in brain regions of continued neurogenesis both in newborn and adulthood, such as dentate gyrus and olfactory bulb, are susceptible to anesthesia-induced apoptosis. These factors might result in a high rate of apoptosis in “normal” control. Due to our parallel group study design, however, such effects should not affect our results.

Evolutionarily, a functional neuron, even if it is infected with virus, would be more favorable than a dead one for the purposes of viral propagation. But, for the host, the results are deleterious because the surviving SeV infected-OSNs lose their odorant sensitivity. This stands in contrast to Influenza virus, a common virus that has always been used in models to explore the relation between virus and OSNs [13]. A recombinant neurovirulent influenza A virus (strain R404BP) induced rapid tissue destruction of olfactory epithelium and apoptosis of OSNs in mice, but did not invade the central nervous systems or kill animals, which suggest induction of apoptosis in OSNs at an early stage of infection might play a positive role in preventing dissemination of neurovirulent influenza A virus into the brain [46]. In contrast, for a highly pathogenic avian influenza (HPAI) H5N1 virus, another subtype of influenza virus, the olfactory system is a major route for brain invasion [47–48]. Thus, the results of these experiments support the concept that neuronal dysfunction rather than neuronal death may play an important role in SeV-caused olfactory disorders, and by analogy, those caused by Parainfluenza viruses in humans. How these results (and those of our studies) relate to PVOD in humans remains to be elucidated.

Of course, the quantity of neurons in the olfactory epithelium is important to maintain olfactory function. However, in clinical studies, there is no a correlation between the number of residual OSNs in biopsies from patients with PVOD and performance on odor threshold and identification testing [49]. Our results may help explain this phenomenon, as SeV caused altered function and decreased apoptosis, but not cytopathic effects, highlighting the complexity of this system. Recently, Gomme observed [50] survival of neurons infected with Rabies virus does not restore them to their pre-infection functionality using gene expression profiles even after viral clearance. This suggests that SeV, a similarly neurotropic virus, may trigger permanent neuronal damage that can persist or progress in the absence of sustained viral antigen and without gross effects. The model described here may allow future work to target the precise etiology of SeV neuropathogenesis in OSNs.

Besides persistent neuronal dysfunction, impairment of regeneration is another important factor that contributes to the persistence of olfactory loss in this model. Neurogenesis has long been recognized as a special property of the adult olfactory epithelium since the1960s [51], helping to maintain critical sensory function after injury, likely related to the system’s vulnerability to damage by toxins, infectious agents, trauma, and environmental exposures. Growing evidence supports that the concept that adult olfactory neurogenesis is a regulated process with many factors involved, such as death of the OSNs, the density of immature neurons, hormonal effects aging, etc. After infection with SeV, both death and birth of OSNs decreased simultaneously, perhaps allowing dysfunctional OSNs to survive longer and not be replaced by new ones in time. Although we did not explore such a complex mechanism behind the reduced proliferation of OSNs after infection, suppression of apoptosis of OSNs, if not fully, at least partially may affect their regeneration. Decreased neurogenesis can be explained, in part, by problems related to these impaired OSNs and the factors that caused their impairment. Studies by Ishimura [52] support this speculation that loss of apoptosis inducing factors result in cell neurogenesis deficits.

Although inflammatory cells were not prominent through the course of infection in our models, inflammation after a virus infection is an important factor that may impact olfactory neuron function indirectly and we cannot exclude such a mechanism. Whether olfactory dysfunction can be attributed to replication of virus and related direct effects or inflammation and immune response induced by viral infection and other indirect effects or both is a complex question. We speculate the SeV has a direct anti-apoptotic effect on OSNs, in contrast to UV-SeV, which seems to have a pro-apoptosis effect. Unfortunately, the quality of the immunohistochemical staining was not adequate enough for quantitative analysis of the number of SeV antigen positive cells. We concede that based on the present study design, it is difficult to distinguish the direct effect and indirect effect of SeV on apoptosis of OSNs. This model may be useful in future work to untangle these mechanisms.

## Conclusions

We report an animal model of PVOD that utilizes a functional assay of olfaction. The results from our study demonstrate impairment of the physiological function of OSNs by SeV *in vivo* and *in vitro*. Further more, we propose that SeV impairs neuronal apoptosis resulting in impaired proliferation of the olfactory epithelium, and causing impaired regeneration, but also results in OSNs with decreased ability to respond to odorants. Further study of this model may allow us to understand the persistence of olfactory loss in patients who can trace their sudden smell loss to an URTI.

## Supporting Information

S1 File Data of proliferation and apoptosis of olfactory receptor neurons. (XLS)
